# Deletion of Metallothionein Exacerbates Intermittent Hypoxia-Induced Oxidative and Inflammatory Injury in Aorta

**DOI:** 10.1155/2014/141053

**Published:** 2014-08-06

**Authors:** Shanshan Zhou, Yonggang Wang, Yi Tan, Xiaohong Cai, Lu Cai, Jun Cai, Yang Zheng

**Affiliations:** ^1^The Center of Cardiovascular Diseases at the First Hospital of Jilin University, 71 Xinmin Street, Changchun 130021, China; ^2^Kosair Children's Hospital Research Institute at the Department of Pediatrics, University of Louisville, 570 South Preston Street, Baxter I, Suite 321B, Louisville, KY 40202, USA; ^3^Chinese-American Research Institute for Diabetic Complication, Wenzhou Medical College, Wenzhou 325035, China; ^4^Department of Pediatrics, The Second Affiliated Hospital & Yuying Children's Hospital of Wenzhou Medical University, Wenzhou 325027, China; ^5^Departments of Radiation Oncology and Pharmacology and Toxicology, University of Louisville, Louisville, KY 40202, USA

## Abstract

The present study was to explore the effect of metallothionein (MT) on intermittent hypoxia (IH) induced aortic pathogenic changes. Markers of oxidative damages, inflammation, and vascular remodeling were observed by immunohistochemical staining after 3 days and 1, 3, and 8 weeks after IH exposures. Endogenous MT was induced after 3 days of IH but was significantly decreased after 8 weeks of IH. Compared with the wild-type mice, MT knock-out mice exhibited earlier and more severe pathogenic changes of oxidative damages, inflammatory responses, and cellular apoptosis, as indicated by the significant accumulation of collagen, increased levels of connective tissue growth factor, transforming growth factor *β*1, tumor necrosis factor-alpha, vascular cell adhesion molecule 1,3-nitrotyrosine, and 4-hydroxy-2-nonenal in the aorta. These findings suggested that chronic IH may lead to aortic damages characterized by oxidative stress and inflammation, and MT may play a pivotal role in the above pathogenesis process.

## 1. Introduction

Obstructive sleep apnea (OSA) has been recognized as a common respiratory disorder with the estimated prevalence of 3–7% in the general populations [1, 2]. OSA is characterized by recurrent episodes of partial or complete collapse of the upper airway during sleep, resulting in repetitive apneas or hypopneas. These obstructive respiratory events can cause episodes of hypoxia and reoxygenation, which are known as nocturnal intermittent hypoxia (IH) [3–5]. Epidemiological studies have revealed independent associations between OSA and coronary [6–9] and cerebral vascular diseases [9–11]. Because chronic IH (CIH) is a prominent feature of OSA [12], there has been a great interest to understand how CIH exposures cause pathological changes of artery [13–17]. Although results of some experimental studies did not support that CIH exposures cause atherosclerosis in mice [13] even after long-term (8.3 months) exposures [18], there is evidence that CIH may induce preatherosclerotic artery damages. Increased carotid intima-media thickness (IMT) has been observed in patients with OSA [19]. There were also studies that revealed independent associations between the hypoxic stress and increased carotid artery IMT [20] in OSA that can be reversed by continuous positive airway pressure therapy (CPAP) [9].

Oxidative stress, endothelial dysfunction, and inflammation have been considered to be key pathophysiologic processes that mediate cardiovascular injury in patients with OSA [5]. Oxidative stress is defined as an imbalance between the excessive production of reactive oxygen or nitrogen species (ROS or RNS) and the reduced antioxidant capacity. ROS and RNS exerted their cellular harmful effects, which can be counteracted by a variety of specific antioxidants [21]. The plasma concentration of an oxidative marker malondialdehyde (MDA) was found to be higher in patients with OSA than in control subjects [22], suggesting a possible role of oxidative stress in the pathogenesis of OSA. It is also known that inflammation and oxidative stress are reciprocal causes and outcomes [23], and oxidative stress is associated with vascular inflammation. Impaired endothelial function has been accepted as a major pathologic process involved in IH-induced vascular alteration, which mainly may result from IH-induced inflammatory response [24]. Indeed, OSA patients had increased plasma levels of proinflammatory cytokines, such as tumor necrosis factor-alpha (TNF-*α*) and interleukin-6, as well as adhesion molecules for leukocyte recruitment, including intercellular adhesion molecules-1 (ICAM-1) and vascular cell adhesion molecule 1 (VCAM-1) [25, 26]. Proinflammatory cytokines and oxidative stress lead to endothelial dysfunction [27] and to formation of fatty streaks, the early stage of atherosclerosis [28]. Inflammatory molecules influence each other in a complex cascade while forming an atheroma [29].

Metallothionein (MT) is a family of cysteine-rich, low molecular weight proteins that could bind to both physiological (such as zinc and copper) and xenobiotic heavy metals through the thiol group of its cysteine residues, which represents about 30% of its amino acid content [30]. Experimental data suggested that MT exerts cellular protection effects not only against metal toxicity, but also against a variety of oxidative stimuli [31, 32]. Our group has shown protective effects of MT in animal models of diabetes [32, 33]. Based on the results of these studies, it seems reasonable to assume that endogenous MT in aorta may also be protective against IH-induced endothelial damages. However, it should be noted that the potential molecular mechanisms in response to oxidative stress caused by CIH in aorta are different from those in diabetes. The CIH mainly induces hydrogen peroxide [34], while in models of diabetes or doxorubicin induced injury, superoxides were predominant mediators that interact with NO to form peroxynitrite [32, 35, 36]. Therefore, whether MT plays a pivotal role in CIH induced aortic damage cannot be simply extrapolated and has to be investigated experimentally. Therefore, using a mouse model of IH to mimic hypoxia-reoxygenation events that occur in OSA patients, we investigated aortic oxidative damage and inflammatory responses in a time-dependent manner during this process, particularly focusing on the potential role of MT.

## 2. Materials and Methods

### 2.1. Animals

MT-KO and WT 129S1 mice were purchased from Jackson Labs. In MT-KO mice (stock number: 002211), both MT1 and MT2 genes were simultaneously disrupted using a vector that inserted in-frame stop codons into the exons of the two genes. Mutant alleles are transcribed but not translated. Six mice were initially used in each group. All animal experimental procedures were approved by the Institutional Animal Care and Use Committee of University of Louisville, which is certified by the American Association for Accreditation of Laboratory Animal Care.

### 2.2. IH Exposures

The murine model of IH exposures during sleep was used in this study as previously reported [37, 38]. Briefly, adult mice were exposed to an IH profile designed to produce similar nadir hemoglobin oxygen saturations (50~60%) and apnea/hypopnea index (AHI: 21–50 times/hour) as observed in moderate to severe OSA patients. The IH paradigm consisted of alternating cycles as 20.9% O_2_/8% O_2_ FiO_2_ (30 episodes per hour) with 20 seconds at the nadir FiO_2_ during the 12 hr light phase. After IH exposures, mice were transferred to room air and sacrificed for tissue collection.

### 2.3. Aorta Preparation and Histopathological Examination

After anesthesia, thoraxes were opened and the descending thoracic aortas were isolated carefully without rips or cuts. Aortic tissues were fixed in 10% buffered formalin overnight. The fixed tissues were cut into ringed segments (approx. 2-3 mm in length) for being dehydrated in graded alcohol series, cleaned with xylene, embedded in paraffin, and sectioned at 5 *μ*m thickness for pathological and immunohistochemical staining.

Histological evaluation of aorta was performed after H&E staining with Image Pro Plus 6.0 software for measuring the tunica media width size as the thickness of aortic tunica media. For immunohistochemical staining, paraffin sections from aortic tissues were dewaxed and incubated with 1X Target Retrieval Solution (Dako, Carpinteria, CA) in a microwave oven for 15 min at 98°C for antigen retrieval, followed by 3% hydrogen peroxide for 10 min at room temperature and 5% animal serum for 60 min, respectively. These sections were then separately incubated with primary antibodies against connective tissue growth factor (CTGF) at 1 : 100 dilution (BD Biosciences, San Jose, CA), transforming growth factor (TGF-*β*1) at 1 : 100 dilution (Santa Cruz Biotechnology, Santa Cruz, CA, USA), tumor necrosis factor-alpha (TNF-*α*) at 1 : 50 dilution (Abcam, Cambridge, MA), vascular cell adhesion molecule 1 (VCAM-1) at 1 : 100 dilution (Santa Cruz Biotechnology, Santa Cruz, CA, USA), 3-nitrotyrosine (3-NT) at 1 : 400 dilution (Millipore, Billerica, CA), 4-hydroxy-2-nonenal (4-HNE) at 1 : 400 dilution (Alpha Diagnostic International, San Antonio, TX), and metallothionein (MT) at 1 : 100 dilution (Dako Inc, Carpinteria, CA) overnight at 4°C. After the sections were washed with PBS, they were incubated with horseradish peroxidase conjugated secondary antibodies (1 : 100–400 dilutions with PBS) for 1 h at room temperature. For color development purposes, immunohistochemical staining sections were treated with peroxidase substrate DAB kit (Vector Laboratories, Inc. Burlingame, CA) and counterstained with hematoxylin to localize the nucleus.

The quantitative analyses of these immunohistochemical staining were achieved from 6 mice of each group. Three sections at interval of 10 sections from each aorta (per mouse) were selected and at least five high-power fields were randomly captured per section. Image Pro Plus 6.0 software was used to transfer the staining density in area of interest to an integrated optical density (IOD), and the ratio of IOD/area in experimental group was presented as a fold relative to that of control.

### 2.4. Sirius-Red Staining for Collagen

Aortic fibrosis was detected by Sirius-red staining of collagen, as described in our previous study [39]. Briefly, sections were stained with 0.1% Sirius-red F3BA and 0.25% Fast Green FCF. The stained sections were then assessed for the presence of collagen using a Nikon Eclipse E600 microscopy system.

### 2.5. Terminal Deoxynucleotidyl-Transferase-Mediated dUTP Nick-End Labeling (TUNEL) Staining

TUNEL staining was performed with formalin-fixed, paraffin-embedded sections using Peroxidase* in situ* Apoptosis Detection Kit S7100 (Millipore, Billerica, MA), according to the manufacturer's instructions. The positively stained apoptotic cells were counted randomly in five microscopic fields at least in each of the three slides from each mouse under light microscopy. The percentage of TUNEL positive cells relative to 100 nuclei was presented.

### 2.6. Quantitative Real-Time PCR (qRT-PCR)

Aortas were frozen with liquid nitrogen and stored at −80°C. Total RNA was extracted using the TRIzol Reagent (Invitrogen). RNA concentrations and purities were quantified using a Nanodrop ND-1000 spectrophotometer. First-strand complimentary DNA (cDNA) was synthesized from total RNA according to manufacturer's protocol (Promega, Madison, WI, USA). Reverse transcription was run in a Master cycler gradient (Eppendorf, Hamburg, Germany) at 42°C for 50 min and 95°C for 5 min with 0.5 *μ*g of total RNA in a final volume of 20 *μ*L that contained 4 *μ*L 25 mM MgCl_2_, 4 *μ*L AMV reverse transcriptase 5x buffer, 2 *μ*L dNTP, 0.5 *μ*L RNase inhibitor, 1 *μ*L of AMV reverse transcriptase, 1 *μ*L of dT primer, and nuclease-free water. Primers of CTGF, TGF-*β*, TNF-*α*, and VCAM were purchased from Applied Biosystems (Carlsbad, CA, USA). The qPCR was carried out in a 20 *μ*L solution including 10 *μ*L of TaqMan universal PCR master mix, 1 *μ*L of primer, and 9 *μ*L of cDNA with the ABI 7300 Real-Time PCR system. Data were expressed as fold increase compared with levels measured in controls by using the ΔΔCt method and *β*-actin as a reference gene.

### 2.7. Statistical Analysis

Data were presented as mean ± standard deviation (SD,  *n* = 6). One-way ANOVA was used to detect the differences between groups, followed by repetitive comparing Tukey's test with Origin 7.5 Lab data analysis and graphing software. Statistical significance was considered as *P* < 0.05.

## 3. Results

### 3.1. MT-KO Mice Exhibited Earlier and More Severe IH-Induced Aortic Pathological Changes and Fibrosis

**Figure 1:**
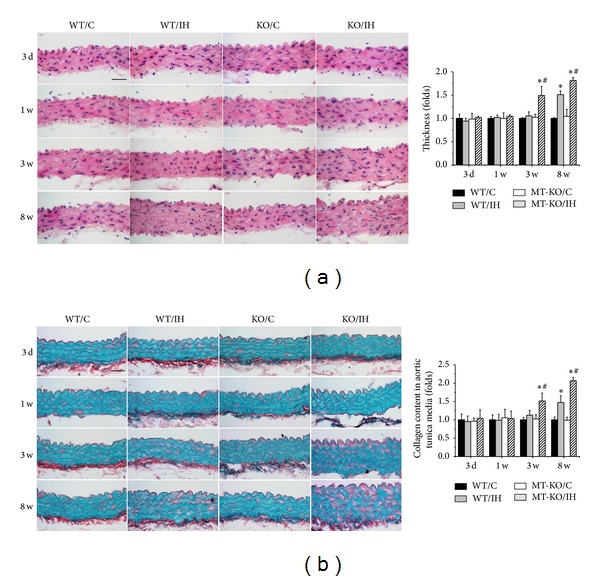
MT-KO mice exhibited earlier and more severe IH-induced aortic pathological changes. The pathogenic changes of aortas were examined by H&E staining (a), and the accumulation of collagen was detected by Sirius-red staining (b), followed by semiquantitative analysis. Data were presented as means ± SDs (*n* = 6); *P < 0.05 versus WT/C; ^#^ *P* < 0.05 versus WT/IH. Bar = 50 *μ*M.

**Figure 2:**
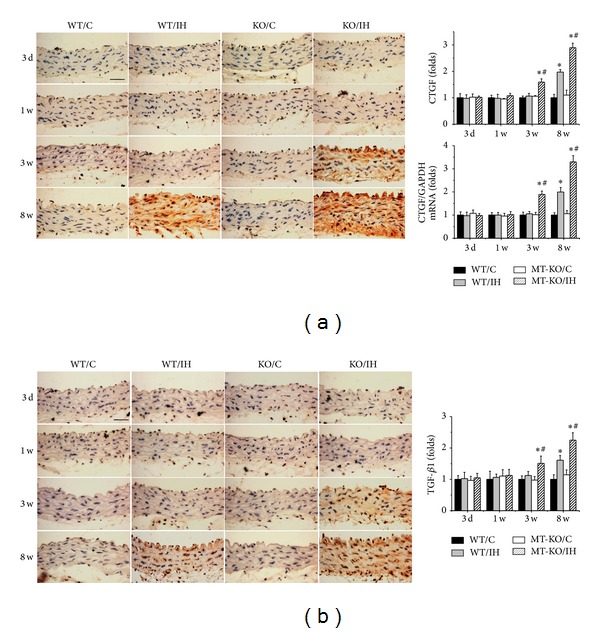
MT-KO mice exhibited earlier and more severe IH-induced aortic fibrosis. Aortic fibrosis was examined by immunohistochemical staining and qRT-PCR for the expression of CTGF (a) and immunohistochemical staining for TGF-*β*1 (b), followed by semiquantitative analysis. Data were presented as means ± SDs (*n* = 6); *P < 0.05 versus WT/C; ^#^ *P* < 0.05 versus WT/IH. Bar = 50 *μ*M.

At the end of experiment, aortas were examined pathologically by H&E staining, which displayed significantly increased tunica media thickness in wild-type mice after exposure to IH for 8 weeks as compared with the room air controls (Figure 1(a)). Sirius-red staining also revealed an increased collagen accumulation in tunica media of aortas by IH exposures for 8 weeks (Figure 1(b)). Moreover, all these pathological changes were observed as early as 3 weeks and exacerbated at 8 weeks after IH exposures in the aorta of MT-KO mice (Figure 1). To further detect the effect of MT on IH-induced aortic fibrosis, immunohistochemical staining and qRT-PCR for both protein and mRNA levels of profibrotic mediators, CTGF (Figure 2(a)) and TGF-*β*1 (Figure 2(b)), were measured. Compared to wild-type mice, aortic CTGF and TGF-*β*1 levels in MT-KO mice were significantly increased after exposures to IH only for 3 weeks, and the differences of CTGF and TGF-*β*1 levels between the two groups were even more remarkable after 8 weeks of IH (Figures 2(a) and 2(b)).

### 3.2. MT-KO Mice Exhibited Earlier and More Severe IH-Induced Aortic Inflammation and Oxidative Damage

**Figure 3:**
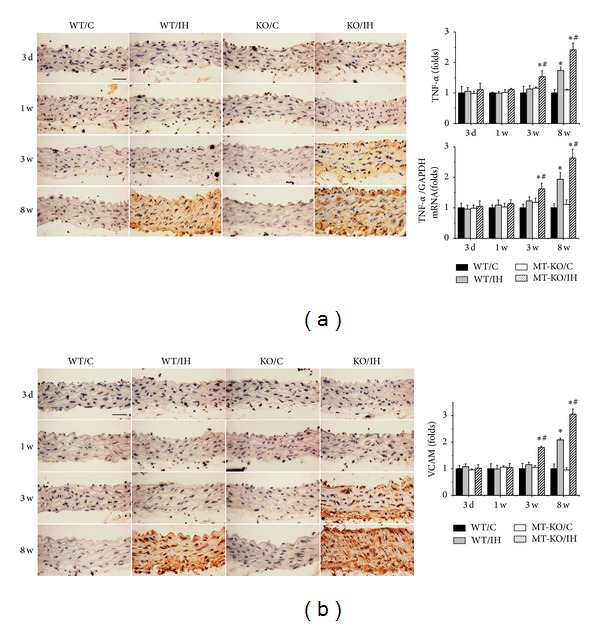
MT-KO mice exhibited earlier and more severe IH-induced aortic inflammation. Aortic inflammation was examined by immunohistochemical staining and qRT-PCR for the expression of TNF-*α* (a) and immunohistochemical staining for VCAM-1 (b), followed by semiquantitative analysis. Data were presented as means ± SDs (*n* = 6); *P < 0.05 versus WT/C; ^#^ *P* < 0.05 versus WT/IH. Bar = 50 *μ*M.

Previous studies have suggested that exposures to IH could induce systemic inflammation, as shown by increased ICAM-1 expression in mesenteric vessels [40] and tumor necrosis factor (TNF)-*α* in lymphocytes from OSA patients [41]. In view of both inflammation and oxidative damages being primary risk factors for the vascular endothelium remodeling, the protein and mRNA levels of TNF-*α* (Figure 3(a)) and VCAM-1 (Figure 3(b)) were examined via immunohistochemical staining and qRT-PCR, which showed that aortic tunica media were significantly increased in wild-type mice after 8 weeks of IH. It was also noticed that expression of TNF-*α* and VCAM-1 was significantly increased in MT-KO mice even exposed to IH for only 3 weeks, which is significantly earlier than those in wild-type mice, and the differences were even more remarkable after 8 weeks of IH (Figures 3(a) and 3(b)).

**Figure 4:**
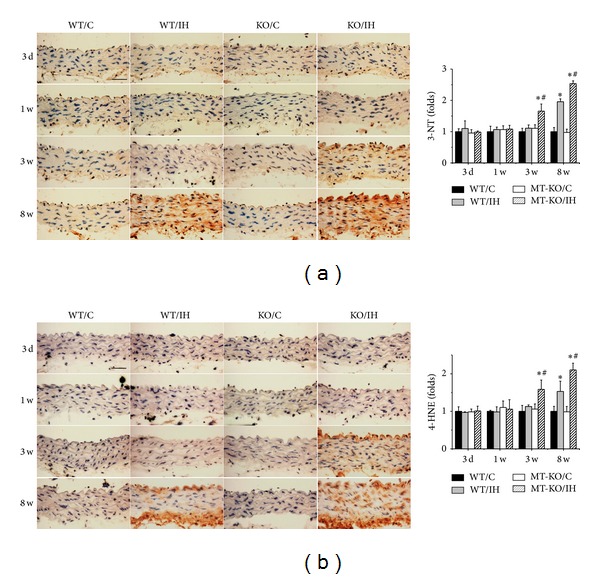
MT-KO mice exhibited earlier and more severe IH-induced aortic oxidative damage. Aortic oxidative damage was examined by immunohistochemical staining for the accumulation of 3-NT (a) and 4-HNE (b), followed by semiquantitative analysis. Data were presented as means ± SDs (*n* = 6); *P < 0.05 versus WT/C; ^#^ *P* < 0.05 versus WT/IH. Bar = 50 *μ*M.

**Figure 5:**
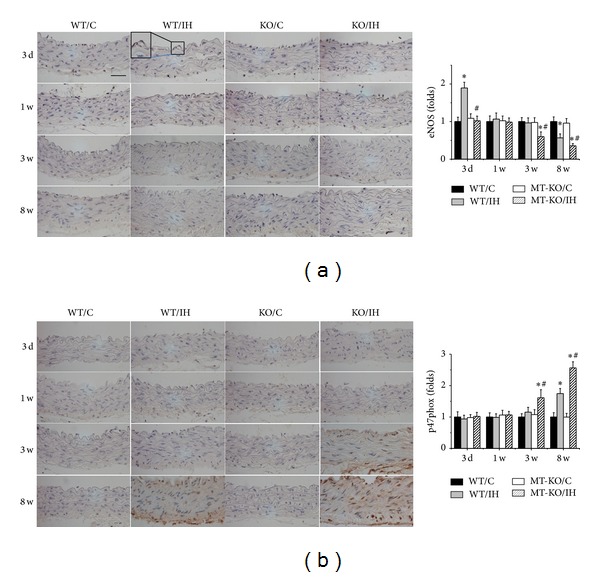
Effects of MT on eNOS and p47phox expression in aorta. Aortic eNOS (a) and p47phox (b) expression were examined by immunohistochemical staining, followed by semiquantitative analysis. Data were presented as means ± SDs (*n* = 6); *P < 0.05 versus WT/C; ^#^ *P* < 0.05 versus WT/IH. Bar = 50 *μ*M.

Since it is well accepted that inflammation and oxidative stress are reciprocal causes and outcomes [23], we went on to examine the markers of oxidative stress in the aortas of each group. Results of immunohistochemical staining showed a significant increase in oxidative and nitrative damage in the aortic tunica media of wild-type mice after 8 weeks of IH, as shown by the accumulation of 3-NT (Figure 4(a)) and 4-HNE (Figure 4(b)). 3-NT and 4-HNE levels were also significantly increased in aorta of MT-KO mice after exposures to IH for 3 weeks, and the differences of 3-NT and 4-HNE levels were even more remarkable after 8 weeks of IH (Figures 4(a) and 4(b)). eNOS expression was upregulated in response to 3-day IH but significantly decreased at 8 weeks of IH in wild-type mice, and MT-KO mice showed no increased of eNOS expression at early stage of IH exposures but further aggravated decrease in eNOS expression after exposures of IH for 8 weeks (Figure 5(a)). P47phox expression significantly increased in aorta of MT-KO mice after exposures to IH for 3 weeks and further increased after 8 weeks of IH compared to wild-type mice (Figure 5(b)).

### 3.3. MT-KO Mice Exhibited Earlier and More Severe IH-Induced Aortic Cell Death

**Figure 6:**
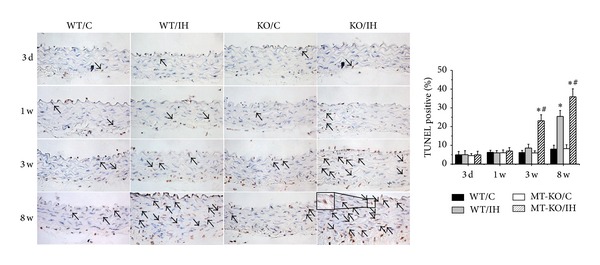
MT-KO mice exhibited earlier and more severe IH-induced aortic apoptosis. The apoptotic cell was examined by TUNEL staining followed by semiquantitative analysis. Data were presented as means ± SDs (*n* = 6). *P < 0.05 versus WT/C; ^#^ *P* < 0.05 versus WT/IH. Bar = 50 *μ*M.

To examine the effect of IH on aortic cell death and the role of MT in IH-induced cell death, we examined aortic apoptosis in MT-KO and wild-type mice by TUNEL staining (Figure 6). The results showed that cell death in aortas of wild-type mice was significantly increased after 8 weeks of IH. Compared to wild-type mice, cell death in MT-KO mice was exhibited earlier after 3 weeks of IH exposures and the differences of cell death between these two groups were even more remarkable after 8 weeks of IH.

### 3.4. Aortic MT Level Was Increased at the Early Stage and Decreased at the Late Stage of CIH Exposures

**Figure 7:**
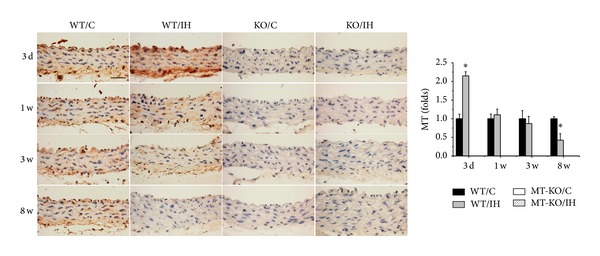
Effects of IH on aortic expression of MT. Aortic expression of MT was examined by immunohistochemical staining (a) with semiquantitative analysis (b). Data were presented as means ± SDs (*n* = 6). *P < 0.05 versus WT/C; ^#^ *P* < 0.05 versus WT/IH. Bar = 50 *μ*M.

Above findings indicated that, in response to IH exposures, MT gene deletion exacerbated IH-induced aortic fibrosis, inflammation, oxidative damage, and apoptosis. So next we detected the dynamic changes of MT protein level in aorta directly. Intriguingly, in wild-type mice, results of immunohistochemical staining showed that there was an early induction of MT expression at the 3rd day of IH, which subsequently returned to normal and then significantly decreased at the 8th week of IH exposures, as compared to controls (Figure 7). No MT expression was detected in aorta of MT-KO mice at each time point independent of whether the mice were exposed to IH or not.

## 4. Discussion

The present study was designed to determine whether aortic expression of MT was changed in response to IH exposures and whether MT deletion exacerbated IH-induced aortic pathological changes. We demonstrated that short-term IH induced the protein level of MT in aorta, while long-term IH inhibited the expression of MT. Moreover, MT levels in aorta seemed to be negatively associated with aortic pathologic damages, including aortic remodeling, oxidative stress, and inflammation, suggesting a possible compensative response of MT induction. By using global MT-KO mice, we further confirmed that deletion of MT aggravated IH-induced aortic pathological changes.

Chronic inflammation plays an important role in the development of various chronic diseases, including OSA [42, 43]. The effects of chronic inflammation include induction of oxidative stress, apoptotic cell death, and endothelial dysfunction, all of which could contribute to the structural and functional abnormalities of the cells [42, 44]. In the present study, we demonstrated the IH induced aortic inflammation, as shown by increased expression of TNF-*α* and VACM-1 in the aortic tunica media after 8 weeks of IH, which was accompanied with increased expressions of markers of aortic oxidative stress (3-NT, 4-HNE), cell death, and remodeling (aorta tunica media thickness, collagen accumulation, and the expressions of CTGF and TGF-*β*1) in IH group. These findings are consistent with the previous concept that inflammation and oxidative stress are reciprocal causes and outcomes [23], both of which are main pathogenic factors for the development of various cardiovascular diseases under stressed conditions. All these pathogenic alterations were exhibited as early as 3 weeks and got more severe after 8 weeks of IH exposures in aorta of the MT-KO mice.

Oxidative stress has been accepted as an imbalance between the excessive production of ROS and the reduced antioxidant capacity, which plays a pathogenic role in the IH-induced hypertension in patients with OSA [45]. This study showed that IH-induced aortic oxidative stress damage closely involved the NO pathway. NO plays a key signal molecule in regulating vascular function; it is generated by NO synthases (NOSs), which comprise endothelial NOS (eNOS), inducible NOS (iNOS), and neuronal NOS [46]. Intimal eNOS-derived NO is transported to the vascular smooth muscle cells of the vascular media and regulates vascular tension mainly by relaxing the vessels, thereby maintaining vascular function [47]. In our study, we showed that eNOS expression was upregulated in response to 3-day IH but significantly decreased at 8 weeks of IH. Compared to their WT controls, MT-KO mice showed no increase of eNOS expression at early stage of IH exposures but decreased at 3 weeks of IH and further aggravated after exposures of IH for 8 weeks. NADPH oxidase is the main source of oxidative stress in the cardiovascular system [48]. Generation of ROS by mitochondria or NADPH oxidase (Nox) may contribute to the altered activity of the carotid chemoreceptor and brain injury in sleep apnea [49, 50]. The expression patterns of the multiple Nox subunits differ between cell types, resulting in different contributions to disease development. ApoE null mice that are deficient in p47phox, a regulatory subunit for both Nox1 and Nox2, have a greater reduction in atherosclerotic lesion size than mice deficient in either Nox1 or Nox2 alone [51–53]. In the present study, we reported that MT deletion exacerbated the IH-induced increased Nox subunits p47phox expression. Taken together, the early upregulation of eNOS expression was assumed as an important compensatory response to induce NO to protect aorta, while downregulation of eNOS and upregulation of p47phox at the late stage may imply the dyscompensatory response so that there was significant increase in oxidative stress and damage.

MT, a potential antioxidant, was found to be increased at the early stage (i.e., at the end of 3-day IH exposures), slightly downregulated at 3 weeks, and significantly decreased at 8 weeks in aorta of wild-type mice. The early induction of aortic MT expression may be assumed as an important compensatory response, while downregulation of MT at the late stage may suggest a decompensatory response, as reflected by the fact that the oxidative stress and damages were significantly increased in the late stages of IH exposures. This hypothesis was supported by the findings that MT-KO mice are more sensitive to CIH-induced aortic damage, as shown by either increased severity of pathologic markers that were observed in both wild-type and MT-KO mice or induction of these markers early only in MT-KO mice. Thus, our study indicated that MT plays a critical role in IH-induced aortic oxidative damage, inflammation response, and vascular remodeling.

One of the special characteristics of MT, as compared with the other antioxidants, is that it can be endogenously expressed in most of the organs and it is also inducible. MTs exist in multiple organs with several molecular forms, including isoforms of MT1, MT2, MT3, and MT4 [32, 54]. A multigene family with at least 14 closely related and pseudogenes encodes MT proteins, and most MT genes, including the functional MT genes (MT1A, MT1B, and MT2A), lie on human chromosome 16 [55]. An earlier study compared the +838 C/G MT2A polymorphism for 288 patients with atherosclerosis and 218 healthy controls showed increased inflammatory cytokines in the patients with atherosclerosis [56]. MT1A gene in rs8052394 SNP is also most likely the predisposition gene locus for diabetes or the changes of serum superoxide dismutase activity [57]. In the present study, we also found deletion of MT aggravated IH-induced aortic pathological changes which may contribute to formation of atherosclerosis by using MT-KO mice in which both MT1 and MT2 genes are deleted. Furthermore, overexpression of MT decreases the sensitivity of pulmonary endothelial cells to oxidant injury [58]. In one of our previous studies, we have also demonstrated that zinc supplementation of diabetic mice induced a significant increase in aortic MT expression, accompanied with significant prevention of diabetes-induced pathogenic changes in the aorta [39]. So induction of MT may be one of the important compensatory components, which protects aorta from chronic IH-induced damages. Therefore, induction of MT may be considered to be clinically applied for patients with OSA to prevent the development of aortic pathological processes induced by CIH exposures.
